# Bilingualism and Development of Literacy in Children: A Systematic Review

**DOI:** 10.11621/pir.2023.0101

**Published:** 2023-03-15

**Authors:** Ekaterina S. Oshchepkova, Natalia A. Kartushina, Ksenia O. Razmakhnina

**Affiliations:** a Lomonosov Moscow State University, Moscow, Russia; b University of Oslo, Oslo, Norway; c Institute of Linguistics, Russian Academy of Sciences, Moscow, Russia

**Keywords:** Bilingualism, multilingualism, biliteracy, writing skills, multilingual education, systematic review

## Abstract

**Background:**

The importance of biliteracy in bilingual children’s development has been widely investigated and discussed for the last several decades, suggesting beneficial effects of writing and reading in two languages for bilingual children as well as for adult second language learners.

**Objective:**

To analyze research on the link between bilingualism and literacy development in two or more languages and the factors that may influence a successful or problematic biliteracy acquisition. RQ (1): What is the relationship between bilingualism and literacy of bilingual children? RQ (2): What strategies are used to develop biliteracy?

**Design:**

The review analyzes 50 studies of literacy development in bilingual children. The selected articles have been separated based on their methodology: 25 articles gave a critical analysis of more than 1,100 studies on the topic, strengthening the theoretical basis of existing research, and 25 other articles were empirical research articles demonstrating practical evidence for the former.

**Results:**

Our analysis revealed that literacy in bilinguals, or biliteracy, can be seen as a necessary condition for fluent development of bilingualism, though it is not a necessary condition (which is explained by the difference between structures of specific languages and writing systems, instruction in literacy, and cognitive baggage invoked by the task used to measure the skill) (Bialystok, 2002). Research suggests that bilingualism impacts children’s ultimate acquisition of literacy via the beneficial effects of bilingualism overall: advanced biliteracy boosts the development of phonological and phonemic awareness and metacognitive abilities. Thus, biliteracy can be considered as an advantage in terms of maintaining bilingual acquisition in general and developing writing skills in particular.

**Conclusion:**

There is a lack of studies on the development of writing skills in different educational contexts, across countries and cultures, which must be addressed and complemented by new empirical research. Research will enable policymakers to improve educational programs in accordance with the needs of bilingual children, who are the majority in the current global population.

## Introduction

Written texts are used as symbolic representations of spoken language. The ability to decode such symbols is an acquired skill. Alkhaldi and Oshchepkova (2018) examined the relationship between speaking and literacy (reading and writing), showing evidence for a tight link between the abilities to encode and decode spoken language, as well as their connection to the development of non-verbal cognition.

Day (2015) and Graham (2019) state that writing ability is acquired through extensive reading (ER). Thus, the key factor for writing skill development for both monolinguals and bi-/multilinguals is exposure to reading materials. Orthographic input can lead to improvements in vocabulary, text organization, and spelling (Murphy & Diehm, 2020). Research focused on writing skills must take into consideration reading skills, since they are tightly linked (Hinesly, 2019; Midgette & Philippakos, 2018).

Writing samples help educators assess not only linguistic characteristics of a written text, but also important aspects of phonemic awareness, phonological awareness, metalinguistic knowledge, and general cognitive abilities (Cheung et al., 2011; Cummins, 2021; Gort, 2019; Rubin & Galván, 2005). In addition, researchers define reading skills as high cognitive abilities, but also as a central part of children’s socialization from the early age (Barletta et al., 2011; Bialystok, 2001; Peng & Kievit, 2020). Therefore, children’s social skills develop not only via speaking, but also via reading and writing acquisition. Literacy skills of bilinguals or multilinguals attract even more attention in research due to the complexity of simultaneous and/or subsequent learning to read and write in two or more languages.

Taking into consideration a considerable range of issues related to multilingual literacy, there is a certain lack of knowledge on the influence of bilingualism on writing skill development and factors affecting literacy development in bilingual children. Most research on bilingual literacy has been performed in natural learning environments, such as schools and academies where children learn reading and writing (Benson, 2017; Cenoz & Gorter, 2011; Evans, 2020; Francis, 1999; Hinesly, 2019). Bilingual education is offered in a number of countries today as an obligatory part of a modern education, with an array of programs offered by different curricula. Despite substantial research on bilingual education and the development of literacy, there are still unresolved issues related to bilingual and/or plurilingual literacy, including their connection to metalinguistic and phonological awareness, non-verbal cognition, cross-linguistic influence, and development of two (or more) writing systems and Cognitive Academic Language Proficiency (CALP) (Cummins, 2021). In addition to research in academic environments, there are also studies focused on the development of biliteracy in home environments, including studies on minority language maintenance and parental strategies to acquire L2 while maintaining L1 (Costa & Melo-Pfeifer, 2022; Griva & Chostelidou, 2014; Kenner, 2005; Lee, 2020; Oller et al., 2007; Reese et al., 2008). These studies are mostly focused on the link between such strategies and bilinguals/biliterates’ academic performance.

The importance of biliteracy in bilingual children’s development has been widely investigated and discussed for the last several decades, suggesting beneficial effects of writing and reading in two languages for bilingual children as well as for adult second language learners. The present study analyzes research on the link between bilingualism and literacy development in two or more languages and the factors that may influence successful or problematic biliteracy acquisition.

RQ (1): What is the relationship between bilingualism and literacy of bilingual children?

RQ (2): What strategies are used to develop biliteracy?

## Methods

### Identification of Relevant Studies

Given the controversy over the effects of bilingualism on the development of writing skills and cognition, the present paper analyzes research on the development of writing skills in bilingual children. We searched studies with the keywords “biliteracy”, “bilinguals”, “writing skills”, and “development”, finding 6,850 articles. Then on the basis of the abstracts, we excluded the studies that were not suitable. The exclusion criteria were the following: (a) studies with adult participants were excluded according to the topic of the research as well as studies with participants who had any specific language impairment (SLI) or studies in which participants lacked the ability to write in L1 when learning to write in L2; (b) studies that repeated the data and the results of earlier research. We have also chosen articles with higher citation by other researchers, as they included a wider analysis than studies that reviewed their conclusions. Fifty articles met our inclusion criteria. So, we analyzed 50 studies on biliteracy development (years of publication are 1979–2022), distinguishing the major topics of interest as presented in Table 1. The selected articles have been separated based on their methodology: 25 articles gave a critical analysis of more than 1,100 studies on the topic, strengthening the theoretical basis of the existing research, and 25 other articles were empirical research articles demonstrating some practical evidence for the former.

**Table 1:** Topics Discussed in Research on Biliteracy.

Major issues the raised research in on biliteracy	Examples of studies	Summary of results
Cognitive skills and brain activity (neurolinguistic studies)	Bialystok (2001); Brice & Brice (2009); Cummins (2021); Del Maschio et al. (2020); Deluca et al. (2019); Ma et al. (2020); Marian & Kaushanskaya (2004); Pliatsikas et al. (2020); Ramírez-Esparza & García-Si- erra (2014); Soltero-González & Butvilofsky, (2016)	In comparison to poor bilingual writers, good bilingual writers held a broader and more complex view of their own writing process and showed more strategic knowledge, since they were more flexible in using both cognitive and metacognitive strategies and employed a wider range of more “elaborated” strategies.
Metalinguistic awareness	Basetti (2005, 2012); Bialystok (2001); D’Angelo, F. (2020); D’Angelo & Sorace (2022); Eviatar et al. (2018); Francis (1999); Kenner (2005); Oller et al. (2007); Robinson et al. (2022); Roehr-Brackin (2018); Tabors et al. (2003)	Biliterates differ from monoliterates in their ability to analyze and manipulate language units, and bilinguals who have developed an aspect of metalinguistic awareness through biliteracy can outperform native speakers in one or both of their languages, including a weak second language. Hence, there is a strong connection between metalinguistic awareness and biliteracy.
Phonological and phonemic awareness	Bassetti, (2005, 2012); Bialys-tok (2002, 2020); Cheung et al. (2011); Gottardo et al. (2011); Khalaf et al. (2019); Limberger et al. (2020); Medeiros et al. (2020); Pawlicka et al. (2018); Tabors et al (2003)	According to the Orthographic Depth Hypothesis, phonological transparency of an alphabetic writing system affects bilinguals’ reading and spelling, as biliterates rely more on grapheme-phoneme conversions for reading and phoneme-grapheme for spelling (i.e., learning to read in two similar writing systems is easier than learning to read in two different writing systems).
Relationship between reading and writing skills	Alkhaldi & Oshchepkova (2018); Bassetti (2012); Bi- alystok (2002); Cheung et al., (2011); Chung et al. (2019); Eviatar et al. (2018); Gottardo et al. (2011); Kenner (2005); Marian & Kaushanskaya (2004); O’Brien et al. (2019)	Bilinguals’ reading proficiency is a joint result of a language’s orthographic characteristics and instructional methods. Reading and writing involve the same processes in all languages, so good readers in one language tend to be good readers in another, as well as being able to spell in both languages. Some language structures and orthography determine the prerequisite skills that children acquire and the ease with which reading can be acquired. Biliterates tend to map a visual stimulus of one language onto an orthographic representation of both languages, and the lexical representation of non-target language is activated (eye-tracking data).
Parental strategies and SES of the families	Brito et al. (2021); Cobo- Lewis et al. (2002); Fernandes (2019); Goldenberg et al. (2011); Griva & Chostelidou (2014); Kenner (2005); Naeem et al. (2018); Nakamura (2018); Reese et al. (2006, 2008); Reese & Goldenberg (2008)	Home literacy practices predicted reading skills in bilingual children. Though the majority of parents were interested in their children’s bilingualism, families with low SES demonstrated a low level of involvement in promoting literacy.
Role of environment (neighborhood, community)	Claussenius-Kalman (2021); Hristo et al. (2017); Oller et al. (2007); Ramos et al. (2022); Reese & Goldenberg (2008); Reese et al. (2006); Tabors et al., (2003); Turdaliyevich (2022)	Bilingualism, when nurtured in well-designed environments of teaching and community, was beneficial for the maintenance of L1 and successful learning of L2. The community’s opinion about languages plays a fundamental role in establishing a child’s self-confidence about bilingualism as well as biliteracy.
Role of education	Barletta et al. (2011); Benson (2017); Carlisle & Beeman (2000); Cobo-Lewis et al. (2002); Elorza (2013); Gale et al. (1981); Goldenberg et al. (2011); Hinesly (2019); Ifont & Tovar-García (2018); Ken- ner (2005); Oller et al., (2007); Serna & Hudelson (1993); Uranova et al. (2022)	The type of bilingual program — immersion (target language only); maintenance (or developmental) bilingual instruction (L1 instruction continues after beginning substantial amounts of L2 instruction); dual-language (or two-way) programs (simultaneous bilingualism and biliteracy in both languages) — affects biliterate acquisition (it can be both positive and negative); school affects students’ confidence and self-identification; teachers’ beliefs about bilingual/biliteral students’ capacities to acquire a subject (there is a prevailing belief that a child’s native language is the cause of his/her learning problems).
L1–L2 transfer in writing	Barletta et al. (2011); Cenoz & Gorter (2011); Cummins (2021); Fitzgerald (2006); Genesee (2002); Kiramba, (2017); Lay (1982); Marian et al., (2021); Soltero-González & Butvilofsky (2016); Reese & Goldenberg (2008); Tabors et al. (2003)	Becoming literate in one’s L1 helps with literacy development in L2 and vice versa: writing well in one’s first language is associated with successful acquisition of writing skills in a second language (due to the process of language transfer).
Personal preferences and self- identification	Butvilofsky, (2016); Denissova et al. (2019); Kiramba (2017); Sabti et al. (2019); Soltero- González, Griva, & Chosteli- dou (2014)	Bilingual children with a low level of support and encouragement from schools and the community did not feel comfortable about their writing difficulties, among other things. Such children completed tasks unwillingly and did not achieve high scores in comparison to monolinguals.

*Note. Languages presented in the studies: English, Spanish, Latin American Spanish, Catalan, Basque, Hakka, Mandarin, Cantonese, Náhuatl, French, German, Arabic, Russian, Gupapuyngu, Albanian, Armenian, Georgian*

## Results

We distinguished nine major topics of research in bilingual literacy development, including cognitive skills and brain activity; metalinguistic awareness; phonological and phonemic awareness; the relationship between reading and writing skills; parental strategies and the socio-economic situation of the families; environmental influence, which includes the influence of the neighborhood and the community; education, including bilingual programs or any other schooling experience in L1 and/or L2 that may influence literacy acquisition in both L1 and L2; L1–L2 transfer in writing, which includes studies focused on cross-language transfer and the interdependence hypothesis by Cummins (2021). The final topic is personal preferences and self-identification, which includes the psychological state of a biliterate and his/ her attitude to “being bilingual and/or biliterate”. Table 1 lists the articles reviewed on these topics and a summary of their results. The *Note* on the languages covered in the reviewed studies demonstrates the breadth of the research, as well as the limitation of existing research (most of the studies dealt with English as one the target languages, rather than other language pairs or multilingual language groups).

## Discussion

### 1. Cognitive Skills and Brain Activity (Neurological Studies)

A critical overview by Barac and Bialystok (2011) showed evidence for a bilingual advantage in cognitive abilities which include executive functions (*e.g.*, attention control, inhibition, task-switching, cognitive flexibility, updating information in working memory). Bialystok (2020) proposes that bilinguals’ cognitive advantage over monolinguals is explained by two factors: (a) bilinguals need to constantly inhibit the linguistic system they are not using at a particular moment; (b) the control, including inhibition, attention, monitoring, and switching, leads to higher development of executive function. Neurological studies have shown that executive control tasks activate the frontal lobe, where Broca’s area is located, the area which is considered as the language-eloquent area and responsible for language processing. Subsequently, bilingualism boosts the development of executive control processes in childhood and adulthood. Older bilinguals show slower cognitive decline than monolinguals in the same processes (Bialystok, 2020).

Brice and Brice (2009), in their research on reading and writing development in bilingual children, identify the differences between brain organization of monolinguals and bilinguals. The organization of language is claimed to vary according to three aspects. The first is the age at which a person becomes bilingual, which is also one of the primary factors influencing bilingual development in general (Brice & Brice, 2009; Grosjean, 1989). Brain imaging research has shown that early biliterates show less separation between language areas in the brain than late biliterates, suggesting that the necessity to accumulate two or more writing systems requires more solid neurolinks between brain areas. This means that the ability to operate more than one language orally and in writing influences the development of brain more heavily. The second aspect is the level of language proficiency, which indicates that more proficient bilinguals show less language separation than less proficient bilinguals. The third aspect that impacts language organization is the brain area itself and the task to be solved. This hypothesis finds support in the study by Chinese researchers Ma et al. (2020), who show that there are significant differences in neural processing related to writing skills in Cantonese–Mandarin bilinguals, depending on the task to be completed in these two languages. These aspects were elaborated in accordance with the Common Underlying Proficiency (CUP) model by Cummins (2021), which postulates that specific neural correlates exist in the bilingual brain and uphold learning of more than one language. Bialystok (2002) concludes that cognitive performance benefits development of advanced literacy, but is not a major factor affecting biliteracy improvement.

### 2. Metalinguistic Awareness

Metalinguistic awareness, as an element of cognition, is one of the primary aspects discussed in studies of bilingualism, especially in terms of bilingual development. Research has shown that bilingual children have more elaborated reading strategies than monolingual children, presumably because of bilinguals’ higher metalinguistic skills (Bialystok, 2001, 2002).

There is evidence that L2 learners read and write in their L2 differently than native monolingual speakers (Bassetti, 2012; Brice & Brice, 2009; Francis, 1999). This difference has been linked to bilinguals’ ability to identify and manipulate linguistic units of two writing systems (Bassetti, 2005). This involves, among other things, bilinguals’ ability to identify linguistic patterns of a specific language and to recognize them in another. For instance, word awareness, knowledge of words as linguistic units, helped bilinguals to recognize even non-existing words in a lexical decision task in the experiments of Bassetti (2005) and Marian et al. (2021). Word awareness enables bilinguals to identify words in a written or spoken text and to single out morphemes or phrases as not being “words”. On the other hand, it has also been demonstrated that literate speakers, both monolingual and bilingual, tend to be aware of the linguistic units represented in the language or languages they use. Thus, users of alphabetic writing systems are aware of phonemes, while users of syllabic writing systems are aware of syllables (Bassetti, 2005). All in all, bilingual ability to distinguish linguistic units in the language used by a person will depend on the type of writing system used in a particular language.

The connection between biliteracy and metalinguistic awareness was also shown by Francis (1999), who compared 45 bilingual students (languages of usage were Spanish and Náhuatl) in the 2nd, 4th, and 6th grades, their language dominance, reading and writing in their L1 and L2, language awareness, and awareness of sociolinguistic relations. The findings aligned with the theory of Cognitive Academic Language Proficiency (CALP) development of bilinguals (Cummins, 2021), which includes linguistic abilities necessary for understanding academic content), stating that literacy-related skills learned in Spanish were not available when the students were asked to read, write, or speak in Náhuatl. However, the developing ability to control language processing on the level of lexical borrowings reflects a conscious awareness of linguistic forms and structures in both languages. Therefore, the period of instruction in both languages must be taken into account for bilinguals as well as L2 learners. Moreover, in writing tasks, participants showed more active manipulations of language representations confirming (and explaining) the connection between biliteracy and metalinguistic awareness. Research based on Hebrew–Arabic speaking bilingual preschoolers and first-graders confirmed this hypothesis (Eviatar et al., 2018).

However, it needs to be mentioned that a bilingual advantage has mostly been shown in control tasks (*e.g.*, tasks ignoring misleading information) rather than in analysis tasks (*e.g.*, explicit knowledge of structure). Bialystok (2001, 2002) determines control of attention as a specific cognitive process that is to be taken into consideration, rather than metalinguistic awareness as a whole.

### 3. Phonological and Phonemic Awareness

Phonological awareness, as another element of cognition entailing explicit analysis of speech, strongly predicts reading abilities of bilinguals (Cheung et al. 2011; Gottardo et al., 2011; Khalaf et al., 2019; Limberger et al., 2020; Medeiros et al., 2020; Oller et al., 2007; Pawlicka et al., 2018). Morais (1991) describes true phonological awareness as a concept including “phoneme-level skills only and the conscious reflection on an abstract representation of speech”. Phonological awareness is also described as a specific metalinguistic ability that develops alongside general metacognitive control processes during middle childhood (Turner, 1991, cited in Gottardo et al., 2011).

Bilingualism is strongly associated with greater phonological awareness (Bassetti, 2012; O’Brien et al., 2014). Bassetti (2012) uses the term Orthographic Depth Hypothesis (ODH), which was introduced by Coltheart et al. (1993) in relation to the impact of phonological transparency in an alphabetic writing system on the capacity to read and spell. According to the ODH, users of phonologically opaque alphabetic writing systems rely more on whole-word units for reading and spelling, whereas users of transparent alphabetic writing systems rely more on graphemephoneme conversions for reading and phoneme-grapheme conversions for spelling (Cotheart et al., 1993, cited in Bassetti, 2012). The research also supports a view on the reading and writing processes of bilinguals as “writing-system-specific”. This is explained by bilingual-specific skills of interpreting a message from one language to the other, which results in written code-switching (Geva & Siegel, 2000, cited in Bassetti, 2012).

The link between bilingualism and stronger phonological awareness was shown by Oller et al. (2007), who compared phonic abilities and vocabulary knowledge, while controlling for socio-economic status (SES), in 620 Spanish–English bilingual children. The results highlighted the leading role of vocabulary knowledge and advanced phonic abilities in reading and writing. Oller et al. (2007) concluded that phonemic awareness of bilinguals is transferred from their L1 to L2, which can significantly benefit L2 reading acquisition if the two languages use a similar alphabetic system (*e.g.*, Spanish and English). Such generalization has been also confirmed by other studies observing cross-language transfer of graphemic-phonemic mappings for Spanish–English bilinguals beginning to read (Cobo-Lewis et al., 2002).

The variation of writing systems has been shown to affect users’ phonological awareness (Borstrom & Peterson, 1998, cited in Cheung et al., 2011; Lundberg et al., 1980; Manis & Freedman, 2001; Muter et al., 1998). This is explained by the specificity of literacy skills as an effortful activity that requires focused instruction over and above the mere availability of written materials (Bertelson, 1986, cited in Cheung et al., 2011). Therefore, bilingual children are able to establish basic concepts for phonological awareness in any language, irrespective of the type of the writing system; therefore, reading can be facilitated for any language in which the initial literacy acquisition occurs (Bialystok, 2002).

However, that may be true only for Indo-European pairs of languages, due to the lack of research in other languages. Phonemes of some Indo-European languages, including English, map directly onto individual letters or letter combinations. Consequently, efficient reading of English and some Indo-European languages relies on phonological knowledge. Researchers have found that phonological awareness of L1 is linked with the ability to read in L2 as well as L2 phonological awareness is related to L2 reading, although these connections are not always completely overlapping (Geva & Wang, 2001). Language-general linguistic knowledge including phonological awareness, may need to be acquired only while acquiring L1 literacy, though, in this case, L1 proficiency would strongly influence L2 acquisition (Flege et al., 1997). Moreover, phonemic awareness was proved to be developed only with alphabetic literacy development, due to the fact that preliterate bilingual children do not show better results than preliterate monolinguals in phonemic awareness tasks (Bassetti, 2012). If there is a scaffolding effect in phonics from Spanish to English, it could minimize or offset any disadvantage that bilinguals might have as a result of the fact that they have to learn two somewhat different systems of phonics (Labov, 2004). However, there might be a disadvantage of bilingualism in terms of phonological and phonemic awareness, since L2 written representations can affect L2 pronunciation, leading to phoneme addition, deletion, and substitution.

### 4. Relationship between Reading and Writing Skills

The current review has shown that most of the studies tend not to separate reading and writing skills, but analyze them as a whole in the form of literacy skills (Brice & Brice, 2009; Carlisle & Beeman, 2000; Cheung et al., 2011; Chung et al., 2019; Eviatar et al., 2018; Francis, 1999; Goldenberg et al., 2011; Kenner, 2005; Marian et al., 2021; O’Brien et al., 2019; Oller et al., 2007; Reese & Goldenberg, 2008; Rubin & Galván, 2005; Tabors et al., 2003). Bialystok (2002) explains this connection by saying that “a crucial preparation for literacy is establishing the forms of the writing systems as symbolic knowledge capable of representing meanings” (p. 178), due to the fact that bilingualism may change the way in which biliterates represent linguistic transformation from oral into written form and vice versa.

According to Bialystok (2002), L2 literacy and the precursors in L1 literacy are interrelated through their common concepts. The first is oral proficiency in the target level, which is important in addition to the general proficiency in either of the languages (both L1 and L2). The second is the representational level for writing concepts that include conscious understanding of writing symbols and their connection with the reality in which the individual’s vision of the world is situated. The third are the metacognitive processes and strategies for reading developed by the individual, which were mentioned above as among the cognitive abilities enhanced by bilingualism.

Cheung et al. (2011) also indicated a tight link between the development of reading and writing skills in bilinguals’ L1 and L2: “reading proficiency is a joint result of orthographic and instructional methods” leading to a cross-language transfer with the transfer of reading to writing skills and vice versa (p. 182). This idea is supported by an empirical study by Carlisle and Beeman (2000), where 36 first-grade students were examined for their reading and writing skills in English and Spanish. Seventeen students were taught in English for the first grade, whereas the other 19 students were taught in Spanish during their first year of education. The children’s text comprehension was assessed by measures of listening and reading comprehension in Spanish and English. Writing samples were evaluated for productivity, linguistic complexity, spelling, and discourse. The study demonstrated the connection between the means and the language of instruction and the development of biliteracy. Instruction in Spanish, as the students’ L1, made a significant contribution to the development of students’ Spanish reading, in comparison to the group who received instruction in English (their L2). Moreover, the Spanish class wrote longer main clauses with greater use of modifiers, and wrote more elaborated stories in Spanish. Thus, the results showed that in terms of bilingual development, progress depends on the language of instruction and on exposure to literacy activities in students’ both native languages (Spanish and English here), as well as the direct connection between (highly-developed) reading and writing skills.

### 5. Parental Strategies and Socio-Economic Status (SES) of the Families

Parental influence on bilingual language acquisition has been repeatedly demonstrated in the literature. One of the most frequently discussed issues was parental input/reaction with respect to children’s code-mixing (*e.g.*, hybridization of two languages, applying units from one language while using the other one). Genesee (2002) suggested the necessity of a “more serious research attention to parental input in the form of bilingual mixing as a possible source of influence in children’s mixing”. However, parental strategies as an influential source of bilingual children’s development are mainly discussed in terms of the development of oral skills or do not differentiate between oral and literacy skills (Brito et al., 2021; Fernandes, 2019; Genesee, 2002; Naeem et al., 2018; Nakamura, 2018). Bilinguals’ parents play an enormous role in building a child’s self-confidence in being bilingual and biliterate. Parental impact on the child’s self-esteem occurs via their awareness of the importance of speaking two (or more) languages, as well as having access to two (or more) cultures. Parents are also responsible for supporting their children’s progress in the two languages, rather than being focused on accuracy and correcting their children’s mistakes. Finally, parents must show their bilingual children that learning and speaking two languages is something positive (Baker & Sienkewicz, 2000).

Therefore, parents primarily establish the child’s bilingual identity and create the environment for bilingual development including his/her literacy skills in both languages. Griva and Chostelidou (2014) examined 32 pupils in Greece (*M* age = 11.4 years, SD = 0.45) from Albanian, Georgian, Russian, and Armenian families who had moved to Greece in their early childhood. The children attended a Greek school where they were learning Greek as their L2 and also English as their foreign language. All of the children were categorized as early bilinguals, while their parents, who also participated in the study, were defined as late L2 learners, as they had spent 2–15 years in Greece. The study inspected writing strategies used by these children while using Greek as their L2, and their parents’ views on their children’s bilingual and biliterate abilities and their attitude to their involvement in their children’s education.

The majority of participants revealed their positive attitude to their biliteracy and ability to express their thoughts in Greek rather than in their L1. Even when the children faced some difficulties in L2 vocabulary or structuring the desirable form of the text, they showed a capacity to adjust the message by making the ideas simpler or less precise or by using a synonym. The participants also commented on their composing process, stating that they were able to generate the ideas while writing in L2 and employing cognitive strategies (*e.g.*, drafting, translating, composing without a draft) simultaneously. Children with poorer writing skills were mostly concerned about correct spelling and using the appropriate vocabulary, whereas children more skilled in writing demonstrated their readiness in the processes of identifying difficulties and self-correction. All participants were aware of their writing problems and used certain compensation strategies to overcome them. The authors connected the abilities to identify obstacles to writing and overcome them with high flexibility in the bilingual children’s use of cognitive and metacognitive strategies and employment of a wide range of more “elaborated strategies’’ (Griva & Chostelidou, 2014).

As for the parental views, the results indicated that although the majority of parents cared about their children’s education, they demonstrated low levels of involvement in it. The authors explain this phenomenon by parents’ low self-esteem, as immigrants. This was particularly experienced by parents who had been living in Greece for less than five years and showed their lack of confidence in using Greek, and unfamiliarity with the school system and culture. The authors also noticed a low level of school support for immigrant families, which might discourage them from getting actively involved in the education of their children (Griva & Chostelidou, 2014).

High influence of the family on biliteral acquisition was also shown by Grunow et al. (2008), who examined 632 Spanish-speaking children in 14 Texas schools (the USA). The authors aimed to understand the relationship between the community and the family in terms of the language used by the children and their literacy opportunities. The results of this study revealed a relationship between the literacy skills in children and their families’ socio-economic status: “access to reading material (aside from what was provided by the school) was limited overall, especially in families living in lower-income areas where few books and magazines were available for purchase in local stores” (Grunow et al., 2008: p. 287). To summarize this line of research, we can conclude that parental attitudes and involvement in their children’s education, and the psychological and economic situation of the family, affect bilingual language acquisition as a whole and biliteral acquisition in particular.

### 6. Role of the Environment (Neighborhood, Community)

As mentioned above, the family plays a crucial role in building the bilingual child’s confidence. The role of the community has been examined mainly in connection with parental strategies that might influence bilinguals’ language development. Reese et al. (2008), in their study of Spanish–English bilingual children in Texas, reported that communities tend to offer a variety of opportunities for children to “hear, see, and use” both English and Spanish (the target language in the study) for a variety of purposes: language use in the community and at home, literacy opportunities in the community, extension of literacy use at home, and the domains of functional literacy uses in which children participate or have an opportunity to observe. Thus, a community with a wide range of bookshops, libraries, churches, events involving the use of literacy skills offers more opportunities for bilinguals to develop their literacy skills, particularly if the authorities are aware of the immigrants’ needs (Reese & Goldenberg, 2008; Reese et al., 2008).

A critical review by Gottardo et al. (2011) suggests that the authorities are responsible for the situation in which immigrants are placed in their territories: “low SES contributes to poor L1, and L2 language and literacy performance”. The authors discuss immigration policy in Canada, as an example of more successful management of immigration flows. In order to move to Canada, a candidate has to provide information about his/her educational status, work experience, personal wealth, as well as knowledge of one of Canada’s official languages. These criteria are decisive when granting a residential permit for potential candidates to move to Canada, which in response, provides a newcomer with the state support necessary for the development of bilingual and biliteral skills.

The results of the analysis indicate that contextual and demographic factors also mediate the relationship between phonological awareness and reading in bilinguals (Gottardo et al., 2011), as well as the educational programs and attitudes towards bilinguals in the community. Family cohesion was shown to mediate the relationship between bilingual dominance and results of a reading test in the study of Latinx immigrant students (Ramos et al., 2022).

### 7. Role of Educational Setting

As mentioned above, the academic setting has a strong effect on the development of writing skills in both monolingual and bilingual students. In terms of multilingual education, Elorza (2013) noted that “teaching more than two languages contributed to schools aiming at building multilingualism and multiliteracy”. Globally, the promotion of multilingual education in schools is growing, calling for more elaborated and approved academic programs. However, taking into consideration bilingual education as a whole, it has been claimed that whatever the method chosen for a specific type of schooling, listening, speaking, reading, and writing are generally taught on equal terms, regardless of the target language or the type of educational program (Elorza, 2013). This observation is in line with the principles of Cummins’s interdependence hypothesis: languages develop interdependently by a transfer of skills and metalinguistic knowledge from one language into another, which is administered by general language competence that does not depend on a particular language (Cummins, 2021).

However, Benson (2017) gives a critical view of the current situation in multilingual education in low-income countries. She criticizes multilingual education for its focus on globally accepted and promoted languages, leaving minority languages aside, which causes a problem of “compromising the recovery and promotion of languages that have been lost or partially lost due to political and/or social repression” (Benson, 2017; p. 106).

As for the diversity of bilingual education, four types of instructional programs for bilinguals are generally distinguished: L2-only instruction (or L2 immersion); early transition (literacy and academic skills are acquired in the home language for the first years of elementary school, then transition to L2-only instruction takes place); maintenance (or developmental) bilingual instruction (beginning and continuing with L1 instruction even after starting to receive substantial amounts of instruction in L2); dual language (or two-way) programs (L1 and L2 speakers receive instruction in both languages — bilingualism and biliteracy are acquired for both groups in both languages) (Goldenberg et al., 2011). Additionally, Content and Language Integrated Learning (CLIL) programs have been shown to play a crucial role in developing L2 academic skills, including L2 acquisition, as well as in maintaining L1.

Cummins (2021), analyzing factors that can influence studies of academic differences in bilingual and monolingual students, distinguished linguistic, socio-cultural and “school program” factors. Teaching approaches in bilingualism have been widely researched (Barletta et al., 2011; Benson, 2017; Carlisle & Beeman, 2000; Forsyth, 2014; Gale et al., 1981; Goldenberg et al., 2011; Hinesly, 2019), and two of them have been discussed more extensively: the dual-language method, which is a two-way bilingual program implementing additive bilingualism; and the method of immersion, implementing subtractive bilingualism (Hinesly, 2019). Speaking of analyzing writing skills in both languages of a bilingual, it is important to look at common characteristics of the written languages such as text structure, organization of content, linguistic functions, and rhetorical resources within a range of text genres (Francis, 1999; Hinesly, 2019). Thus, the characteristics of a story, letter, or essay presented in one language will be applied and will evolve further in other languages of a bilingual/ multilingual. Subsequently, exposure to different text genres — not to mention common characteristics relating to text structure, organization of content, functions, and rhetorical resources within the text, regardless of the language in which the exposure occurs — will be ultimately implemented throughout the linguistic background of an individual (Elorza, 2013).

This type of bilingual instruction was illustrated by the quasi-experimental study by Hinesly (2019), who compared writings of monolingual and bilingual students at the University of Texas (Spanish and English). Importantly, the writings were not evaluated for content but for mechanics, clarity, language, and the whole structure of the text. The result was that monolinguals outranked bilinguals in structure and organization, but bilinguals outperformed monolinguals in grammar and language use. This supports previous research on general bilingual advantage in such areas as grammar usage, ability to determine appropriate content, and language command. Moreover, Hinesly (2019) concluded that the method of immersion in bilingual education (L2-only focused) is unsatisfactory in comparison to fully bilingual approaches that support fluent bilingualism. Consequently, via a correctly chosen teaching approach, students develop plurilingual competence, which is proficiency in multiple languages with a capacity to efficiently use appropriate language to fulfil the tasks necessary in each specific context, including academic ones (Hinesly, 2019).

Another study of writing skills acquisition in bilingual programs was conducted by Gale et al. (1981). The study included 27 students from two bilingual programs: 15 students received literacy instruction in their L1 first (Gupapuyngu), then began receiving instruction in their L2 (English); 12 students from a submersion group received all their instruction in L2, without building L1 literacy. When examining writing samples of the participants, the authors found that those students who had received L1 literacy instruction before switching to L2 instruction wrote papers of higher quality than those in the submersion group. This paper raises some questions on how the children were instructed, in order to understand what other factors might have influenced the results. Still, this study raises the necessity for more detailed research on the inter-relationship between bilingual instruction and development of writing skills.

Generally, people learn the writing system of their language at an early age in primary school, but this may differ for bilinguals and multilinguals. Kenner (2005) and Hinesly (2019) distinguish culturally different approaches to teaching handwriting in English, Chinese, Arabic, Spanish, and other languages, specifically in languages with differing scripts. As far as teaching bilinguals to use different writing systems is concerned, Kenner (2005) suggests that “bilingual children are considered flexible learners who make good use of their learning experience”: they transfer academic skills learned in one language to another, making connections and using all their linguistic resources to express a thought. Thus, the suggestion that writing is impossible without reading is true, and while learning to write, an individual learns to read. On the other hand, learning to read does not necessarily involve writing, but it does involve acquaintance with writing systems represented by a certain language (Bassetti, 2012). Therefore, through a well-chosen and established curriculum, a bilingual or a multilingual can successfully develop academic and bi-/multiliterate skills.

### 8. L1-L2 Transfer in Writing

Bialystok (2002) found that bilingualism can play an important role in learning to read if language-specific skills transfer across languages, because some linguistic structures and orthography, in particular, determine the prerequisite skills to be acquired (Geva & Siegel, 2000, cited in Bialystok, 2002; Goswami, 1999). Therefore, biliterate children acquiring the same script in both languages will find it easier to read and write in their languages rather than biscriptal children learning to use two different scripts for their languages (Bialystok, 2001, 2002; Soltero-González & Butvilofsky, 2016).

Cenoz and Gorter (2011) reported “multi-directionality in language transfer” when comparing multilinguals’ writings in Spanish, Basque, English, and French. Multilingual learners use their languages in multiple directions, transferring not only from L1 to L2, but also from L2 to L1. Furthermore, multilinguals tend to use the same general strategy of writing and focus on the same themes to approach the task, but they tend to use different modes of communication in informal writing, encouraging multi-modal literacy. The authors concluded that real literacy practices need to include “trans-languaging, code-mixing and code-switching” since it is desirable to benefit from all the languages an individual uses, so that what is learned in one language can be easily transferred to (an)other language(s) as well (Cenoz & Gorter, 2011).

Code-switching and code-mixing are considered to play an important role in the acquisition of bilingual writing skills. The effects of cross-linguistic transfer, within-language transfer, and cross-linguistic influence have been recently discussed by Marian et al. (2021), who suggested that cross-linguistic influences on initial vocabulary learning could have “cascading effects on the makeup of one’s later vocabulary” (p. 2). During the acquisition of new vocabulary, a learner is affected by the languages he/she knows, and the initial similarity of some words or patterns can have dynamically changing consequences over the course of word learning as well as usage, both in oral and written communication.

### 9. Personal Preferences and Self-Identification

Research has shown a relationship between the development of bilingual competence and the psychological state of a bilingual (Griva & Chostelidou, 2014; Kiramba, 2017; Soltero-González & Butvilofsky, 2016). Positive attitudes to the ability to control two or more languages reflect on the more elaborated and freer usage of the languages. Ferris and Politzer (1981) analyzed writing samples of 60 bilingual (English and Spanish) students from California. One-half of the participants had begun U.S. public instruction from the very first year of primary education, whereas the other half had been born and begun their education in Mexico. The results showed that despite some insignificant differences in writing abilities in their L2, the participants from the second group did not possess any demonstrably lower English skills than the first group. Moreover, the students had more positive attitudes toward school and school achievement, and seemed more highly motivated in the schools where school achievement and teachers were highly valued. Thus, the self-esteem of a student, both monolingual and bilingual, is affected by school and educators.

The influential role of schools and teachers in the development of bilingualism was discussed above; we notice that they also play an important role in self-esteem. A biliterate with higher self-confidence will more likely use his/her linguistic resources to express himself/herself, whether in written or oral communication. Cenoz and Gorter (2011) carried out research with 165 secondary students learning Spanish or Basque as their L1 and Basque or Spanish as their L2, correspondingly, and English or French as their L3. Although Cenoz and Gorter discussed the role of code-switching in the writing samples of the participants, they also found that multilingual students who identify themselves as competent users of two or more languages are able to encourage multimodal literacy. This was specifically noticed in terms of language mixing in informal writing, when the students used not only different languages in one piece of writing, but different models of communication to make the message more emotional and stronger (Cenoz and Gorter, 2011). It may be therefore concluded that self-identification as a bilingual and/or biliterate boosts a bilingual’s self-esteem in fluent and autonomous language use.

The importance of personal preferences in writing for biliteracy development was also illustrated in a study by Kiramba (2017), which examined writing practices of bilingual students in a rural school in Kenya. In this study, 28 multilingual students, speaking one or two more other languages in their families and/or neighborhoods, were learning Kiswahili and English at school. The multilingual community and school instruction in English made the use of more than one language valuable both orally and in writing. The results of the study indicated that “bi/multilinguals’ learning is maximized when they are allowed and enabled to draw from their previously acquired language skills rather than being constrained and inhibited from doing so by monolingual instructional assumptions and practices” (Hornberger, 2005, p. 607). The participants’ self-esteem was heightened by this, and as was shown in the writing samples, the participants meshed semiotic resources for their identities and interests (Canagarajah, 2013). Consequently, psychological well-being was linked with school achievements, which were supported by the community and family; a combination of these factors affected bi/multilinguals’ self-identification and language development and biliteracy in particular.

In our critical review, one controversial issue about the relationship between bilingualism and literacy remained unexplored. This is the negative effect of bilingualism on literacy acquisition. The view that bilingualism has a negative effect was predominant until the middle of the 20th century (for details, see Zinchenko et al., 2019). In recent years, there has been some evidence that such an effect takes place (Brzdęk & Brzdęk, 2021). However, our analysis shows that a negative effect is observed mainly in children with SLI and other difficulties, and SLI is among the exclusion criteria for our review. Besides, Bialystok says that “there is no evidence for harmful effects of bilingual education and much evidence for net benefits in many domains” (Bialystok, 2018, p. 666). Thus we considered it possible in this review to avoid a detailed consideration of a negative effect of bilingualism.

## Conclusion

Literacy, as a metacognitive process and socio-cultural concept in general, affects how people analyze and manipulate language units represented in writing system(s) in order to achieve their communicative purpose. Written language provides a permanent graphic representation of a language, by segmenting a message into patterns and depicting some aspects of a language that are not present in its spoken modality. Moreover, preliterate bilingual children are no better than preliterate monolingual children in their phonemic awareness, though even very young children are able to differentiate their writing systems and describe how they work (Bassetti, 2012; Kenner, 2005). Biliterates tend to mix their writing systems in order to achieve a humorous effect or to affirm their identity. What is important to remember for schools and academies aimed at promoting multilingual education, is that biliteracy can affect both L2 and L1 production and comprehension.

Thus, the beneficial effects of biliteracy depend primarily on the relationship between the bilingual’s writing systems. Evidence has been found that biliterates are mostly facilitated when their two writing systems are similar (Bassetti, 2012). In accordance with the Interdependence Hypothesis and the BIA+ model (the upgraded version of the Bilingual Interactive Activation model developed by Dijkstra & Van Heuven, 2002, and used for understanding the process of bilingual language comprehension, which, according to the model, consists of the word identification subsystem and task/decision subsystem), the writing and reading processes of the L1 writing system affect L2 reading and writing, since literacy skills in one writing system can be used and are more likely to be used when reading and spelling in the other system (Dijkstra et al., 2019; Dijkstra & Van Heuven, 2002; Jacquet & French, 2002; Koda & Zehler, 2007; Van Heuven et al., 1998).

Therefore, biliteracy might benefit the harmonious development of bilingualism, but is not a sufficient condition (due to the structure of specific languages and writing systems, exposure to instruction in literacy, and cognitive skills) (Bialystok, 2002). Bilingualism inevitably impacts children’s ultimate acquisition of literacy, due to the beneficial effects of bilingualism as a whole, so that advanced biliteracy boosts the development of phonological and phonemic awareness and metacognitive abilities. Thus, biliteracy can be considered as an advantage in maintaining L2 acquisition in general and developing writing skills in particular.

## Limitations

We need more studies on the development of biliteracy concerning languages other than English, and other language pairs with different writing systems and/or scripts. There is an obvious lack of studies related to the development of writing skills in different educational contexts, which must be fulfilled and extended with new research. This will give educational programs an opportunity to improve their curricula in accordance with the needs of bilinguals who are becoming the majority of the global population.
